# Comparative Analysis of Reconfigurable Platforms for Memristor Emulation

**DOI:** 10.3390/ma15134487

**Published:** 2022-06-25

**Authors:** Margarita Mayacela, Leonardo Rentería, Luis Contreras, Santiago Medina

**Affiliations:** 1 Faculty of Civil and Mechanical Engineering, Research and Development Directorate, Technical University of Ambato, Ambato 180207, Ecuador; lf.contreras@uta.edu.ec (L.C.); wsmedina@uta.edu.ec (S.M.); 2 Faculty of Engineering, National University of Chimborazo, Av. Antonio José de Sucre, Riobamba 060108, Ecuador; leonardo.renteria@unach.edu.ec

**Keywords:** memristor emulator, electronic circuit, FPAA, FPGA

## Abstract

The memristor is the fourth fundamental element in the electronic circuit field, whose memory and resistance properties make it unique. Although there are no electronic solutions based on the memristor, interest in application development has increased significantly. Nevertheless, there are only numerical Matlab or Spice models that can be used for simulating memristor systems, and designing is limited to using memristor emulators only. A memristor emulator is an electronic circuit that mimics a memristor. In this way, a research approach is to build discrete-component emulators of memristors for its study without using the actual models. In this work, two reconfigurable hardware architectures have been proposed for use in the prototyping of a non-linearity memristor emulator: the FPAA (Field Programing Analog Arrays) and the FPGA (Field Programming Gate Array). The easy programming and reprogramming of the first architecture and the performance, high area density, and parallelism of the second one allow the implementation of this type of system. In addition, a detailed comparison is shown to underline the main differences between the two approaches. These platforms could be used in more complex analog and/or digital systems, such as neural networks, CNN, digital circuits, etc.

## 1. Introduction

To date, three fundamental passive elements have been used to design electronic circuits: resistors, capacitors, and inductors. In 1971, Leon Chua from the University of California at Berkeley reasoned from symmetry arguments that there should be a fourth fundamental element, which he named memristor [1]. This element is named a memristor, as it combines the behavior of a memory and a resistor. A Memristor is a two-terminal element whose resistance depends on the magnitude, direction, and duration of the applied voltage [2].

In theory, its memory capacity should be infinity, but even if it is not capable of storing energy, it can be used to store information indirectly.

The most important feature is that this device lets us store information in an analog way, leaving us with the possibility to develop applications that we could not even imagine some years ago. Since the fabrication of the Hewlett Packard (HP) memristor [3], interest in memristor research has increased [4]. Several people have started to take advantage of the memristor capacities in different fields, such as analog applications [5,6,7,8,9,10], digital circuits and memories [11,12,13], and the neuromorphic field because of its analog storing capability [14,15,16,17,18,19,20,21,22,23,24,25,26].

Although there is a commercial memristor now available [27], research based on this device in the market has been rare [28], probably due to cost and technical issues, such as voltage and current limitations, measurement, and others [29]. In this sense, the research continues, overall, on circuit modeling [30,31,32] or numerical modeling [33] only to simulate memristor behavior. The modeling and simulation phases are very important for the design of a system or a device [34], but they also need to verify and validate the model in the physical world.

Therefore, there is a need for a physical module that works like a real memristor, that is, a memristor emulator. This memristor emulator is an electronic circuit that mimics a memristor. Hardware implementations of the memristor emulator have been developed, and different models have been proposed. Some of them use digital and analog mixed circuits [35], where a microcontroller acquires the voltage applied to the memristor; it calculates and updates the resistance value by setting the digital potentiometer to obtain the required resistance value. This model is relatively simple to implement, but the time response and resolution of the memristance are limited by the A/D and D/A converters and the digital potentiometer. It has also been implemented as a pure analog model [5], which follows pretty fine the memristor behavior, the connection with other circuit elements is difficult, and the memristance is not guaranteed for a long time. Another emulating circuit [14] shows the features of the memristor; it keeps the memristance constant and stable over a long period of time, and it is compatible with other circuit devices. It has a small variation range of the memristance and a limited ratio between the maximum and minimum values of memristance. Additionally, electrical devices, such as light-dependent diodes [36], light-dependent resistors [37], and junction field effect transistors [38], have been used for this type of implementation despite their small memristance variation range.

Moreover, recent trends in hardware design have seen a strong increase in the use of programmable devices [39], such as CPLDs and FPGAs [40,41], and more recently, field programmable analog arrays (FPAA) [42]. Programmable devices can provide flexible and efficient platforms. These devices satisfy the performance, cost, and power requirements of most hardware prototyping architectures [43]. Reconfigurable architectures combine some of the flexibility of software with the high performance and speed of hardware.

In this paper, we propose a completely different method to emulate the memristor, an analog reconfigurable hardware architecture based on FPAA, and a digital reconfigurable hardware architecture based on FPGA. These include some important features of any memristor emulator [14]. The easy programming and reprogramming of the first hardware architecture and the performance, high area density, and parallelism of the second one allow the implementation of this type of system [44,45].

The principal difference when compared to ordinary hardware emulator implementation is the ability to make substantial changes. It is possible to adapt the hardware during runtime by “loading” a new circuit on the reconfigurable architecture, and there is a significant increase in the number of emulated devices.

Additionally, knowing that there are different models of memristors with an analog pure architecture, completely different circuits for each model must be built to test them. Moreover, with microcontroller-based architecture, it is easier to obtain by changing the software; however, the speed, accuracy, and number of emulated devices are always limited by the rest of the electronic components.

Then, the use of this technology will allow an increase in design complexity while allowing a decrease in the amount of time spent debugging, wiring, and other hardware implementation problems.

## 2. Materials and Methods

### 2.1. Memristor Overview

**Figure 1:**
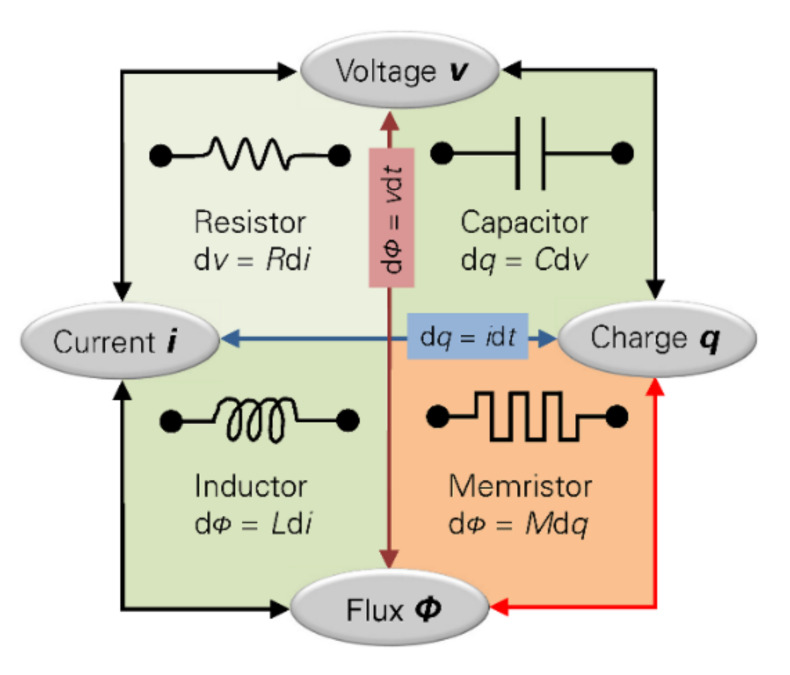
Electronic components relationship.

The conception of memristor as the fourth fundamental component in circuit theory creates a new approach to nonlinear circuit design. As is known, circuit elements (Figure 1) reflect relationships between pairs of the four electromagnetic quantities of charge, current, voltage, and magnetic flux [46].

Resistance relates voltage and current (d*v* = *R*d*i*), capacitance relates charge and voltage (d*q* = *C*d*v*), and inductance relates flux and current (d*φ* = *L*d*i*), respectively [47].

The memristor keeps the last resistance value when it is turned off and retrieves the value when it is turned back on. Memristor has several interesting properties, including pinched hysteresis and dynamic negative resistance, which can have a significant impact on nanoelectronics [2].

A memristor is characterized by its memristance (M) and memductance (W). These are described by the charge-dependent and flux-dependent equations, respectively, as follows [10]:

(1) $$M\left(Q\right)=\frac{d\emptyset \left(q\right)}{dq}$$

(2) $$W\left(\emptyset \right)=\frac{dq\left(\emptyset \right)}{d\emptyset }$$

This property is similar to the fundamental element resistor, which is characterized by its resistance R [48]. It may be noted that memristance is similar to variable resistance. A nonlinear version of Ohm’s law can be expressed as a current-controlled memristive system [49]:

(3) $$V_{m}\left(t\right)=M\left(x,I_{m},t\right)I_{m}\left(t\right)$$

(4) $$\frac{dx}{dt}=f\left(x,I_{m},t\right)$$

where *x* is a vector representing n internal state variables, *Vm*(*t*) and *Im*(*t*) denote the voltage and current across the device, and M the memristance. Similarly, a voltage-controlled memristive system [49]:

(5) $$I_{m}\left(t\right)=G\left(x,V_{m},t\right)V_{m}\left(t\right)$$

(6) $$\frac{dx}{dt}=f\left(x,V_{m},t\right)$$

where G is the memductance.

One important aspect to keep in mind about the memristor is its dependence on the “state” variable x. The state variable describes how the system “looks” inside [13].

**Figure 2:**
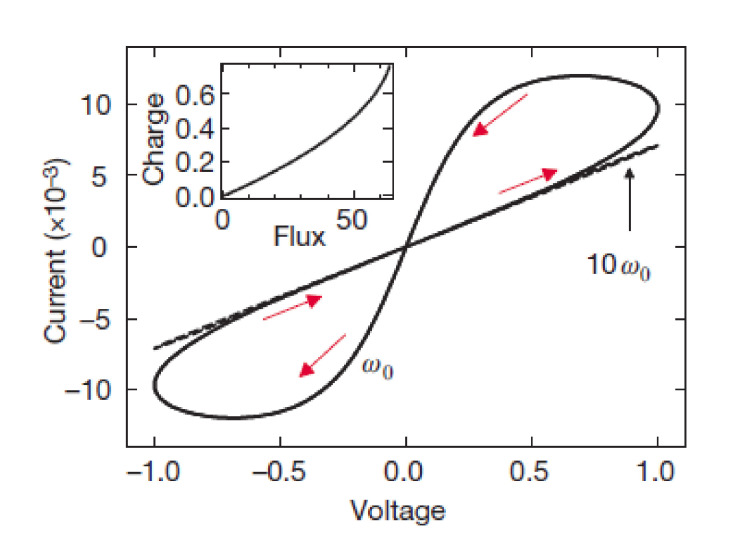
Typical behavior of memristors.

A memristor, which is a two-terminal circuit element, will provide hysteresis loops in an i–v plot when subject to an alternating voltage signal [48]. The hysteresis loops are very valuable when memristive systems are to be identified, and the loops normally run through the origin in an I–V plot (Figure 2).

In the case of linear elements in which *M* is a constant, memristance is identical to resistance, and thus, it is not of special interest. However, the most valuable functions of circuits are attributable to their non-linear characteristics. If *M* is itself a function of q, yielding a nonlinear circuit element, then the situation becomes more interesting [3]. The compatibility of memristors with integrated circuits could provide new circuit functions at extremely high two-terminal device densities [2].

In Different published, mathematical memristor models are used as a baseline to research device features [32].

Memristors exist in various types depending on how they are built; moreover, there are systems that exhibit properties of memristors and so are called “memristive systems” [2].

If we consider a flux-controlled memristor described by Equation (2), where $q\left(\emptyset \right)$ is a smooth continuous cubic function of the form [10]:

(7) $$q\left(\emptyset \right)=-\alpha \cdot \emptyset +\beta \cdot \emptyset^{3}$$

With *α*, *β* > 0. As a result, in this case, the memductance $W\left(\emptyset \right)$ is provided by the following expression:

(8) $$W\left(\emptyset \right)=\frac{dq\left(\emptyset \right)}{d\emptyset }=-\alpha +3\cdot \beta \cdot \emptyset^{2}$$

Since $\emptyset \left(t\right)$≜∫ $v_{m}\left(t\right)dt$, an expression of the current through the memristor with cubic nonlinearity is [49]:

(9) $$i_{m}\left(t\right)=\left(-\alpha +3\cdot \beta \cdot {\left(\int v_{m}\left(t\right)dt\right)}^{2}\right)\cdot v_{m}\left(t\right)$$

However, a certain methodology for how to experiment with the calculation of the memristance function and obtain the memristor flux-charge characteristic has not been defined [50,51]; therefore, there is a need to calculate its memristance. One method might be applying a DC or AC voltage, measuring its current and voltage, and then, by taking the integration of its current, its charge or memristance, as a function of the current, can be calculated [52,53].

### 2.2. Circuit Implementation

Numerical simulation plays an important role in analyzing systems and predetermining design parameters prior to their physical realization [54]. Before implementation, a memristor model was constructed in a Matlab environment. The backward Euler method [55] was used for numerical integration in which the incoming sample was multiplied by the sampling time and added to the last result of the integrator as follows:

(10) $$\frac{dy}{dt}=f\left(t,y\right)\;\;\;\;;\;\;y\left(t_{0}\right)=y_{0}$$

(11) $$y_{n+1}=y_{n}+hf\left(t_{n+1},y_{n+1}\right)$$

As, ∅´≜$v_{m}\left(t\right)$ we can rewrite Equation (10) as follow:

(12) $$\emptyset_{n+1}=\emptyset_{n}+hf\left(t_{n+1}\right)$$

the term $y_{n+1}$ disappears because the equation only depends on the function in time; therefore, the problem is limited to solving the integral of the input voltage, squaring the result, and calculating the current.

**Figure 3:**
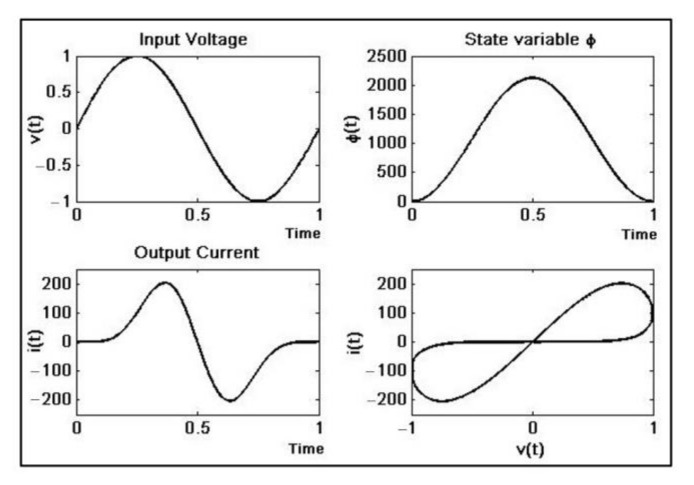
Memristor time response simulation. *v*(*t*) = 1sin(2π*ft*), *f* = 60 Hz, *h* = 0.05, *α* = 0.677 × 10^−3^, *β* = 0.029 × 10^−3^.

After testing and tuning, the numerical simulation results (Figure 3) can be used as a reference for real implementation. Notice the unrealistic values of the current; for that reason, the set of equations requires rescaling in the physical implementation.

#### 2.2.1. FPAA Emulator

The Field-Programmable Analog Array is an integrated circuit that can be configured to implement various analog functions [39]. This is the analog equivalent of FPGA [56]. This electronic device has a feature that can be used to programmatically change component values and interconnections; in other words, it can be dynamically reconfigured. Additionally, FPAA provides more efficient and economical solutions for analog dynamic system designs [45].

The most important elements in an FPAA are the Configurable Analogue Blocks (CAB), which manipulate the signals and the interconnecting routing network. Each element contains configurable modules (CAMs) [39].

The analogue blocks have parameters that can be programmed to accommodate according to the application [39].

**Figure 4:**
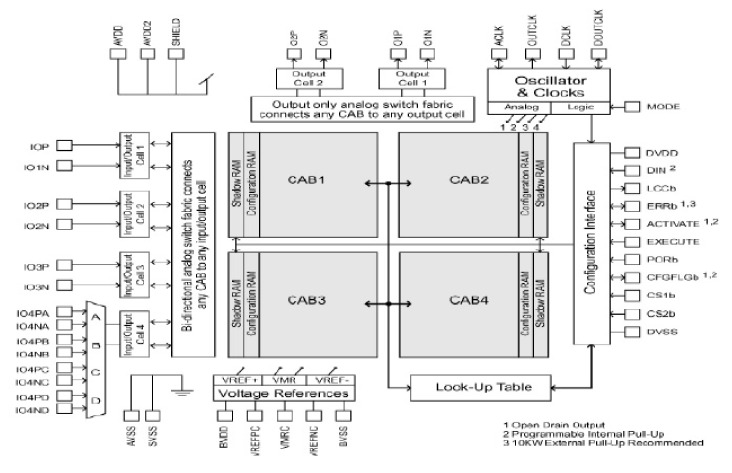
Anadigm AN221E04 Architecture.

The device used is the anadigm AN221E04 (Figure 4), which is composed of four programmable blocks. The dynamic range of the signals in the FPAA device is bounded by physical constraints [56]. In effect, the FPAA device has ±3 V saturation level, so the signal magnitude has to be scaled according to a previous numerical simulation.

**Figure 5:**
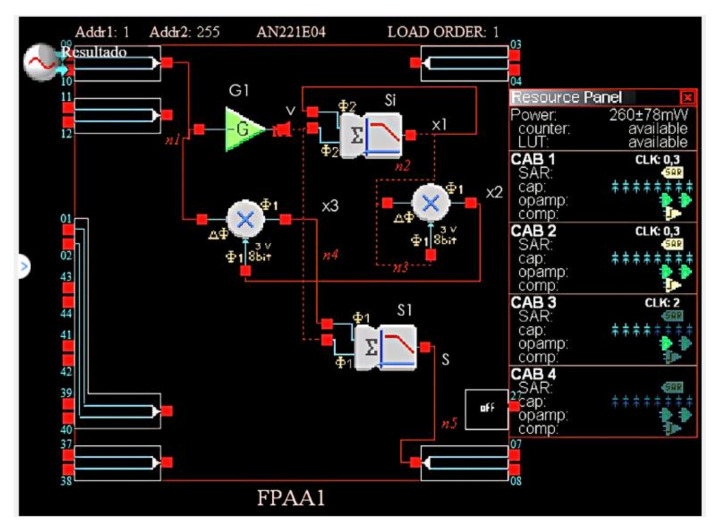
Schematic of FPAA memristor emulator.

The complete system (Figure 5) is composed of a gain inverter, two analog multipliers, and two sum-filter modules (Table 1).

The first stage of the system is the gain inverter G1, by applying an input voltage $v_{m}$, its output v is:

(13) $$v=-\frac{v_{i}}{100}$$

The sumfilter module Si has and output *x*_1_ described as:

(14) $$x_{1}={\dot{x}}_{1}+hv$$

Notice that ${\dot{x}}_{1}$ refers to the last value of $x_{1}$. Then, similar to Equation (10), it is possible to convert Equation (14) to:

(15) $$x_{1}≅\int v\;dt\;x_{1}=\int -\frac{v_{i}}{100}\;dt=-\frac{1}{100}\int v_{i}\;dt$$

After the first multiplier M1, we can obtain $x_{2}$,

(16) $$x_{2}=x_{1}\cdot x_{1}=\frac{1}{100^{2}}{\left(\int v_{i}\;dt\right)}^{2}$$

Now, at the output of the second multiplier M2, we have $x_{3}$,

(17) $$x_{3}=x_{2}\cdot v_{i}=\frac{1}{100^{2}}{\left(\int v\right)}^{2}\cdot v_{i}$$

Finally, the sum filter module S1 gives the following result:

(18) $$S=G_{1}\cdot x_{3}+G_{2}\cdot v$$

With $G_{1}$= 0.87 and $G_{2}$ = 0.0677, and by arranging the terms, we can obtain:

(19) $$S=0.87\cdot \frac{1}{100^{2}}{\left(\int v_{i}dt\right)}^{2}\cdot v_{i}-0.0677\cdot \left(\frac{v_{i}}{100}\right)\;=0.087\times 10^{-3}\cdot {\left(\int v_{i}\;dt\right)}^{2}\cdot v_{i}-0.0677\times 10^{-3}\cdot v_{i}\;S=\left(-0.677\times 10^{-3}+3\cdot 0.029\times 10^{-3}\cdot {\left(\int v_{i}\;dt\right)}^{2}\right)\cdot v_{i}$$

By replacing parameters similar to [5], *α* = 0.677 × 10^−3^, *β* = 0.029 × 10^−3^ and $v_{i}=v_{m}\;$on Equation (19), the result is as follows:

$$i_{m}\left(t\right)=\left(-\alpha +3\cdot \beta \cdot {\left(\int v_{m}\left(t\right)dt\right)}^{2}\right)\cdot v_{m}\left(t\right)$$

**Figure 6:**
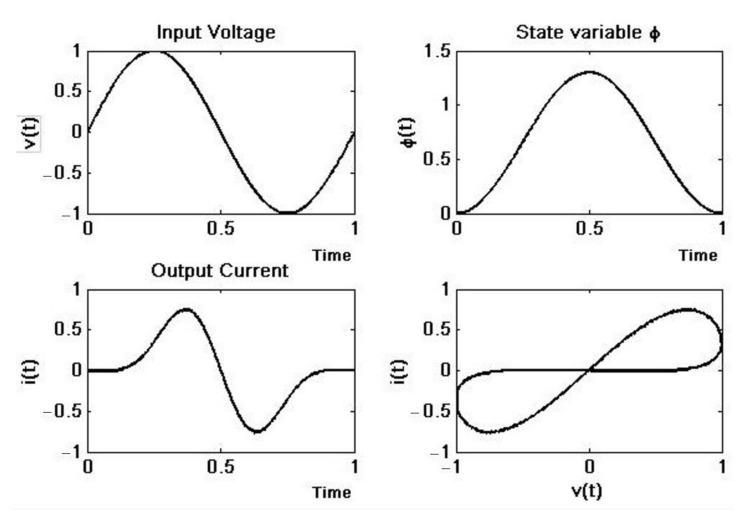
FPAA memristor emulator response.

By applying a sinusoidal signal $v\left(t\right)=v_{o}\cdot \sin\left(2\pi ft\right)$ with *f* = 60 Hz, $v_{o}$= 1 V, we can obtain the $v_{m}$ and $i_{m}$ plots and it’s clear that this element really has memristor behavior (Figure 6).

Since AN221E04 performs analog “calculations” at a certain clock frequency, the values of all settings are limited to a range of values determined by the clock frequency. However, the values can be adjusted by a minimum of 0.01 units, which is acceptable for our needs. Moreover, there are only four CABs for each chip; consequently, an emulator uses almost 50% of the device’s available resources.

#### 2.2.2. FPGA Implementation

The second implementation was based on FPGA architecture. Traditionally, analog computers use operational amplifiers to implement basic operations (addition, subtraction, multiplication) and compute time-integral functions [57]. All of these operations can be performed on the FPGA, except computing time integrals. To solve this problem, a device called a Digital Differential Analyzer (DDA) was created to simulate the integral function digitally.

**Figure 7:**
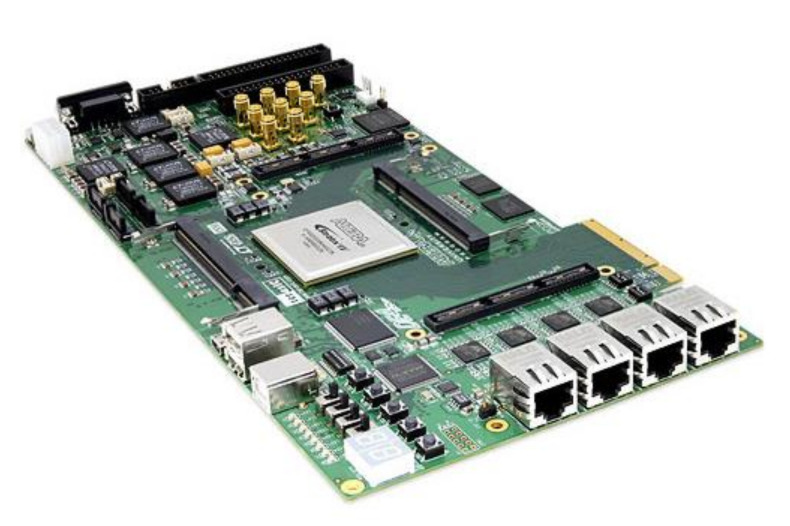
Altera DE4-320 FPGA Development Board.

A Terasic DE4-320 Development Board (Figure 7) was chosen for this implementation. The board is equipped with an Altera Stratix IV GX EP4SGX230 FPGA that provides about 228,000 logic elements (LEs), 91,200 adaptive logic modules (ALMs), 14.283-Mb embedded memory, and eight phase-locked loops. The DE4 Development Board provides the ideal hardware platform for system designs that demand high performance, serial connectivity, and advanced memory interfacing [41]. Developed specifically to address the rapidly evolving requirements in many end markets for greater bandwidth, improved jitter performance, and lower power consumption [58].

A fixed-point version of the DDA was implemented with 32-bit number representation. Bit 32 is the sign bit, and the binary point is between bits 16 and 17 (with bit zero being the least significant) (Table 2). The number range is thus −32,768 to +32,768, and the smallest value to be represented is 1.5259 × 10^−5^.

**Figure 8:**
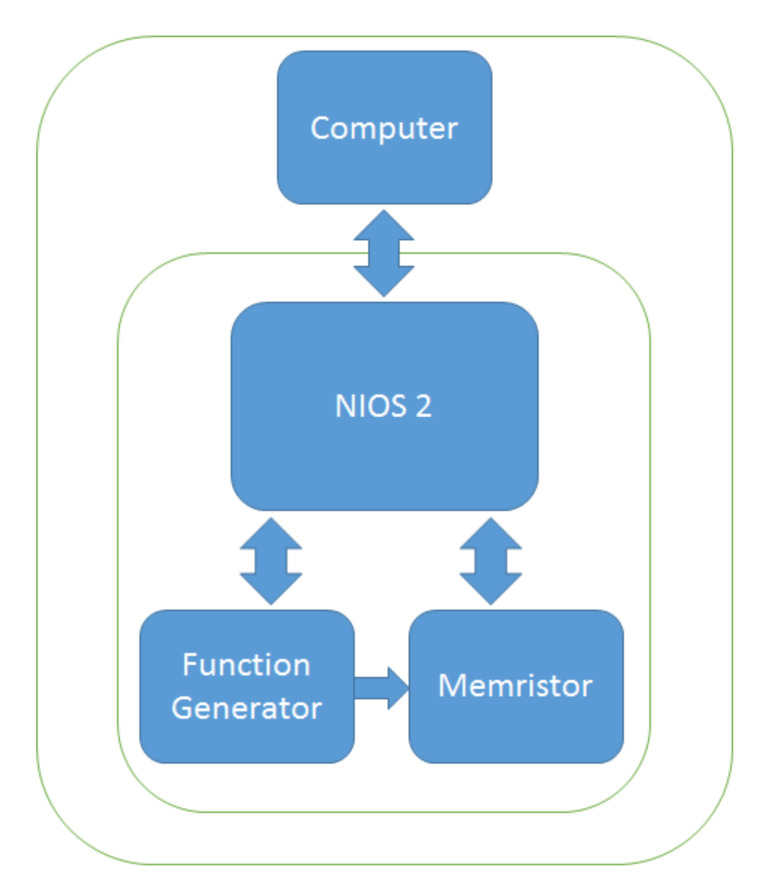
FPGA memristor emulator general diagram.

The device is composed of three principal modules (Figure 8): the function generator, which contains the input signal, the memristor solver that describes the memristor behavior and the NIOS2 IP core, which is used for control and configuration.

The hardware implementation is associated with VHDL programming. The “ieee proposed” HDL package [59] was used to obtain a synthesizable fixed-point unit (FPU).

**Figure 9:**
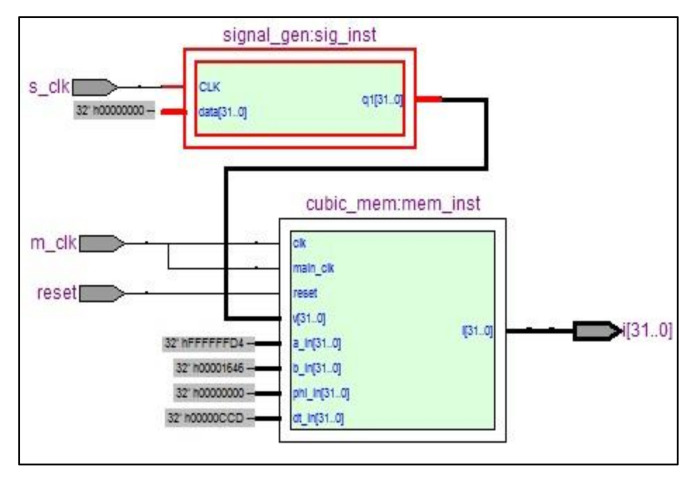
FPGA memristor emulator RTL schematic.

The package allows float and fixed-point operations to be performed by adding, subtracting, and multiplying. The VHDL multiplier is easily implemented; it has three inputs, one of which can be used for scaling. The adding and subtracting operations were calculated directly. The forward Euler method was used for the integration steep; the step size used was 0.005. The function generator (Figure 9) is used to provide various waveforms, such as square wave, triangle wave, sine wave, etc. These waves are generated and stored in a 32 × 256 Ram memory. Intermediate values were interpolated at an 8 MHz sampling rate.

**Figure 10:**
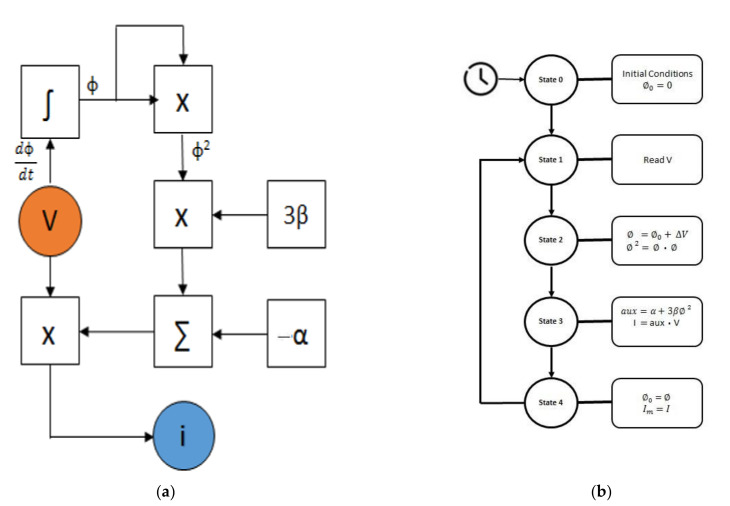
FPGA-based memristor solver: (**a**) Memristor solver block diagram; (**b**) Memristor solver FSM flowchart.

The memristor solver (Figure 10) consists of an FSM that solves Equation (6). It takes four cycles of its 8 MHz clock to solve each sample; other parameters are set by default and could be changed by the Nios 2 core.

**Figure 11:**
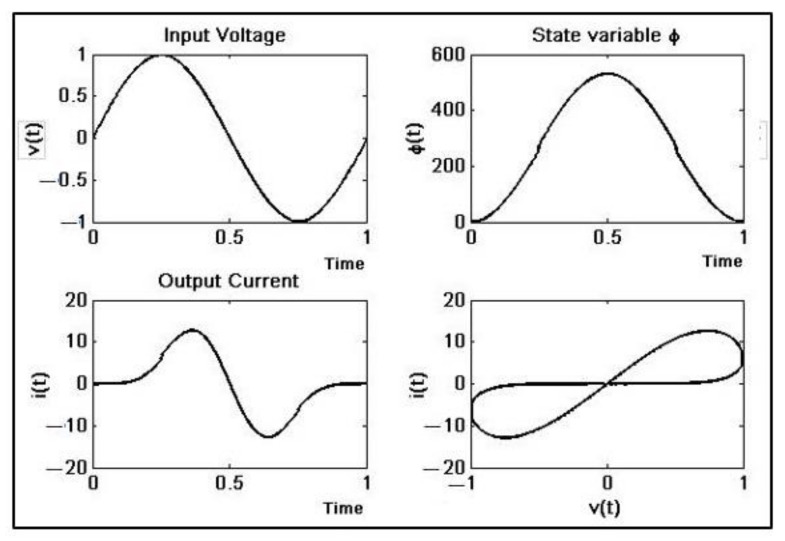
FPGA memristor emulator response.

With parameters similar to [5] *α* = 0.677 × 10^−3^ and *β* = 0.029 × 10^−3^, a signal frequency of 60 Hz, and a sampling frequency of 2 MHz, we can obtain the *v_m_* vs. *i_m_* plots (Figure 11); clearly, the results show the memristive behavior of the system.

The EP4SGX230KF40C2 FPGA chip place and route process statistics (Table 3) give us a general idea of the resources used and the potential capacity of this kind of implementation.

Since the memristor solver is independent, it means that it can work by itself; it is possible to have more memristors managed by the same NIOS 2 core. This gives the possibility of significantly increasing the number of memristors because used resources (Table 4) decrease considerably.

## 3. Results and Discussion

**Figure 12:**
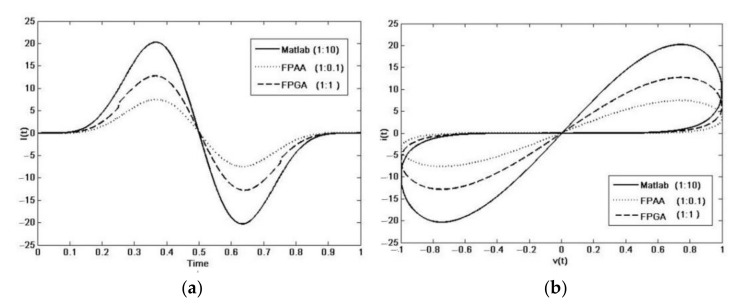
Memristor emulators’ current: (**a**) Current vs. Time plot; (**b**) Voltagevs. Current plot.

The results (Figure 12) show waves similar to those of the simulation, except for an attenuation factor. Losses are due to the switched capacitor technology, and filtering within the FPAA provides a reduction of 99.65%. Otherwise, the FSM within the FPGA integrates 1/4 of the total samples because it needs 4 clock cycles, resulting in an attenuation of 96% with respect to the original wave. In both cases, the attenuation can be used as the scaling factor needed to obtain current values according to the reality of the electronic circuits. This factor can easily be modified to suit the requirements of the external peripherals in any memristor-based circuit implementation.

The time for one integration step depends on the main clock frequency on the FPAA, and on the sampling clock on FPGA at 4 MHz and 2 MHz, respectively (Table 5).

Notice that, on simulation, the time for one period is proportional to the number of samples. On the other hand, in the case of hardware implementation, the total time depends on the signal period (Table 5). It is possible to increase the number of samples by increasing the sampling frequency while the time remains. However, the maximum sampling frequency is limited by the physical constraints, overall, on the FPAA device.

## 4. Conclusions

In this work, two different emulator models were proposed. First, a simple FPAA programmable analog circuit has replaced dozens of standard discrete components. It has become an effective solution to problems of rapid prototyping and has simplified the task of designing similar electronic circuits. However, resources are limited, and at most, it is possible to obtain two emulators for each CI. Likewise, a powerful tool called DDA has given us the possibility of performing a digital integrator on an FPGA, allowing a relatively high number of elements regarding the occupied area density; however, analog interaction is not possible directly, and it will require additional features to obtain this capacity. The next step is to use them as a part of more complex systems, such as chaotic oscillators, voltage-controlled sources, or neural network circuits. Finally, since the discovery of the memristor, researchers have used mathematical models, simulations, and emulators of memristors and, more recently, commercial memristors in their experiments. Simulations are useful but do not consider all factors, especially physical ones; emulators mimic real memristor behavior; however, they are not practical to use in more complex systems such as a neural network due to their design and physical proportions, while the new commercial memristors are still too expensive and make their use difficult, especially when there are budget limitations. Therefore, the memristor emulators proposed in this work become viable alternatives, especially when the applications demand a large number of them.

## Figures and Tables

**Table 1:** FPAA emulator parameter settings.

Name	Options	Parameters	Clocks
Multiplier 1 (Multiplier v1.2.2) 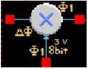 Anadigm (Approved)	**Sample and Hold** Off **Y Input Full Scale** 3 Volts	**Multiplication Factor** 1.00	**Clock A** 125 kHz (Chip Clock 3) **Clock B** 2 MHz (Chip Clock 0)
Multiplier 2 (Multiplier v1.2.2) 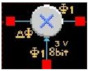 Anadigm (Approved)	**Sample and Hold** Off **Y Input Full Scale** 3 Volts	**Multiplication Factor** 1.00	**Clock A** 125 kHz (Chip Clock 3) **Clock B** 2 MHz (Chip Clock 0)
SumFilter 2 (SumFilter v1.1.3) 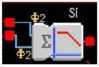 Anadigm (Approved)	**Output Changes On** Phase 2 **Input 1** Non-inverting **Input 2** Non-inverting **Input 3** Off	**Corner Frequency [kHz]** 12.5 **Gain 1 (Upper Input)** 0.870 **Gain 2 (Lower Input)** 0.0676	**Clock A** 125 kHz (Chip Clock 3)
G1 (GainInv v1.1.4) 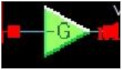 Anadigm (Approved)		**Gain** 0.0100	**Clock A** 2 MHz (Chip Clock 0)
SumFilter 2 (SumFilter v1.1.3) 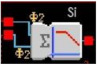 Anadigm (Approved)	**Output Changes On** Phase 1 **Input 1** Non-inverting **Input 2** Non-inverting **Input 3** Off	**Corner Frequency [kHz]** 100.0 **Gain 1 (Upper Input)** 1.00 **Gain 2 (Lower Input)** 0.0500	**Clock A** 1 MHz (Chip Clock 2)

**Table 2:** Fixed-point 32-bit number representation.

Number	Binary	Hexadecimal
1.0	0_000000000000001.0000000000000000	“00010000”
0.025	0_000000000000000.0000011001100110	“00000666”

**Table 3:** FPGA chip statistics, including the full system.

FPGA Resource	Used Resources
Combinational ALUTs	10,345
Total Registers	10,345
DSP Block 18-bit Elements	118
Total Block memory bits	4,434,148
Logic utilization	9%

**Table 4:** FPGA chip statistics memristor solver only.

FPGA Resource	Used Resources
Combinational ALUTs	1298
Total Registers	16,105
DSP Block 18-bit Elements	118
Total Block memory bits	100,352
Logic utilization	1%

**Table 5:** Time performance comparison.

	Fs	One Period Time	
	MHz	Nro Samples	Time
Matlab	8	133,366	64.6 ms
FPAA	4	66,667	16.7 ms
FPGA	2	32,347	16.7 ms

## Data Availability

The data presented in this study are available on request from the corresponding author.
